# Supplementary material for: Seed Weight as a Covariate in Association and Prediction Studies for Biomass Traits in Maize Seedlings
Source: Plants (Basel). 2020 Feb 20;9(2):275. doi: 10.3390/plants9020275 (PMC7076456; doi:10.3390/plants9020275)
Supplement: Supplementary file 1 [file plants-09-00275-s001.zip › plants-704714-supplementary-V4/Supplementary Table S2.docx]

**Supplementary Table S2**. SNPs from MLM+Q+K without HKW crossing arbitrary threshold of 4 associated with biomass traits fresh weight in grams (FW), dry weight in milligrams (DW) and dry matter content as % of FW (DMC) in control (C) and water withholding (WW) treatments. SNPs in bold are the ones crossing Bonferroni corrected threshold value for significance at α=0.05.

| **QTL** | **Trait** | **TRT** | **Marker** | **Chr.** | **Pos.(MBp)** | **-log(P)** | **R2** | **SNP** |
| --- | --- | --- | --- | --- | --- | --- | --- | --- |
| QFWww1 | FW | ww | S10_139734834 | 2 | 17.341 | 4.068 | 4.65 | C/A |
| QFWww2 | FW | ww | S2_176241695 | 2 | 181.463 | 4.055 | 4.81 | T/C |
| QDWc1 | DW | c | S3_12297359 | 3 | 11.583 | 4.236 | 5.37 | G/A |
| QDWww1 | DW | ww | S10_139734834 | 2 | 17.341 | 4.829 | 5.78 | C/A |
| QDWww2 | DW | ww | S3_15473839 | 3 | 14.789 | 4.421 | 5.27 | T/C |
| QDWww3 | DW | ww | S3_16305542 | 3 | 15.433 | 4.288 | 5.26 | A/G |
| QDWww4 | DW | ww | S3_17033783 | 3 | 16.414 | 4.577 | 5.38 | C/T |
| QDWww5 | DW | ww | S3_17613984 | 3 | 16.964 | 4.024 | 4.71 | C/A |
| QDWww6 | DW | ww | S9_14021178 | 9 | 13.709 | 4.102 | 5.18 | T/C |
| **QDMCc1** | **DMC** | **c** | **S1_8741690** | **1** | **8.775** | **6.584** | **9.33** | **C/G** |
| QDMCc2 | DMC | c | S1_34204183 | 1 | 34.541 | 4.516 | 5.93 | C/T |
| QDMCc3 | DMC | c | S1_37203165 | 1 | 37.582 | 4.232 | 5.11 | A/G |
| QDMCc4 | DMC | c | S1_37207054 | 1 | 37.586 | 4.518 | 5.55 | A/G |
| QDMCc5 | DMC | c | S1_37215825 | 1 | 37.594 | 4.284 | 5.13 | A/T |
| QDMCc6 | DMC | c | S1_101643332 | 1 | 103.985 | 4.208 | 5.17 | C/T |
| QDMCc7 | DMC | c | S1_173422581 | 1 | 175.378 | 4.03 | 4.91 | T/C |
| QDMCc8 | DMC | c | S1_295988910 | 1 | 301.48 | 4.401 | 5.31 | G/A |
| QDMCc9 | DMC | c | S2_2805417 | 2 | 2.802 | 4.068 | 4.78 | T/C |
| QDMCc10 | DMC | c | S2_6191374 | 2 | 6.146 | 4.456 | 5.35 | C/T |
| QDMCc11 | DMC | c | S2_7183324 | 2 | 7.092 | 4.207 | 5.11 | G/A |
| QDMCc12 | DMC | c | S3_189463222 | 3 | 192.36 | 4.461 | 5.35 | C/G |
| QDMCc13 | DMC | c | S6_127195 | 6 | 0.177 | 4.165 | 5.18 | C/T |
| QDMCc14 | DMC | c | S6_370986 | 6 | 0.392 | 4.096 | 4.83 | C/T |
| **QDMCc15** | **DMC** | **c** | **S6_8833007** | **6** | **9.248** | **6.596** | **9.07** | **G/C** |
| **QDMCc16** | **DMC** | **c** | **S6_95602988** | **6** | **98.45** | **6.342** | **8.41** | **G/C** |
| QDMCc17 | DMC | c | S6_99136681 | 6 | 101.971 | 4.948 | 6.36 | G/A |
| QDMCc18 | DMC | c | S7_176216182 | 7 | 181.799 | 4.097 | 4.82 | C/A |
| QDMCww1 | DMC | ww | S1_168415551 | 1 | 170.174 | 4.081 | 4.89 | A/G |
| QDMCww2 | DMC | ww | S6_99127885 | 6 | 101.962 | 4.403 | 5.66 | A/C |
| QDMCww3 | DMC | ww | S6_130004982 | 6 | 134.089 | 4.3 | 5.24 | C/T |
