# Supplementary material for: Seed Weight as a Covariate in Association and Prediction Studies for Biomass Traits in Maize Seedlings
Source: Plants (Basel). 2020 Feb 20;9(2):275. doi: 10.3390/plants9020275 (PMC7076456; doi:10.3390/plants9020275)
Supplement: Supplementary file 1 [file plants-09-00275-s001.zip › plants-704714-supplementary-V4/Supplementary Table S3.docx]

| **Parameter** | **Predictive ability** | | | **RMSEP** | |
| --- | --- | --- | --- | --- | --- |
|  | **No cov.** | **HKW** | **No cov.** | | **HKW** |
| FW | 0.49 (-0.40–0.96) | 0.60 (0.06–0.92) | 0.04 (0.02–0.06) | | 0.04 (0.02–0.06) |
| FWww | 0.54 (-0.18–0.96) | 0.39 (-0.61–0.92) | 0.03 (0.01–0.04) | | 0.03 (0.01–0.05) |
| DW | 0.54 (-0.25–0.94) | 0.63 (0.05–0.90) | 3.05 (1.59–4.57) | | 2.89 (1.8–4.45) |
| DWww | 0.60 (-0.10–0.96) | 0.56 (-0.46–0.93) | 2.52 (1.34–4.29) | | 2.64 (1.44–4.27) |
| DMC | 0.53 (-0.48–0.97) | 0.47 (-0.38–0.93) | 0.15 (0.08–0.28) | | 0.16 (0.09–0.29) |
| DMCww | 0.62 (-0.16–0.96) | 0.53 (-0.32–0.91) | 0.28 (0.14–0.47) | | 0.30 (0.17–0.47) |

**Supplementary Table S3:** Predictive abilities and root mean square error of predictions (RMSEP) with ranges across 500 folds of cross validation (in brackets) for FW, DW and DMC in C and WW
